# FDDM1 and FDDM2, Two SGS3-like Proteins, Function as a Complex to Affect DNA Methylation in Arabidopsis

**DOI:** 10.3390/genes13020339

**Published:** 2022-02-12

**Authors:** Shengjun Li, Weilong Yang, Yunfeng Liu, Guangyong Li, Xiang Liu, Yaling Liu, James R. Alfano, Chi Zhang, Bin Yu

**Affiliations:** 1 Key Laboratory of Biofuels, Shandong Provincial Key Laboratory of Energy Genetics, Shandong Energy Institute, Qingdao Institute of Bioenergy and Bioprocess Technology, Chinese Academy of Sciences, Qingdao 266101, China; 2 Center for Plant Science Innovation, University of Nebraska-Lincoln, Lincoln, NE 68588-0666, USA; weilong.yang@huskers.unl.edu (W.Y.); yunfengliu_bio@126.com (Y.L.); gli3@unl.edu (G.L.); xiang_liu@hotmail.com (X.L.); lylzpf@126.com (Y.L.); zhang.chi@unl.edu (C.Z.); 3 School of Biological Sciences, University of Nebraska-Lincoln, Lincoln, NE 68588-0118, USA; 4 State Key Laboratory for Conservation and Utilization of Subtropical Agro-Bioresources, College of Life Science and Technology, Guangxi University, Nanning 530004, China; 5 Department of Plant Pathology, University of Nebraska, Lincoln, NE 68588-0722, USA; 6 Shanghai Chenshan Plant Science Research Center & Botany Garden, Chinese Academy of Sciences, Shanghai 201602, China; 7 College of Life Science, Shanxi Agricultural University, Jinzhong 030801, China

**Keywords:** DNA demethylation, SGS3-like protein, FDDM1 and FDDM2, epigenetics, Arabidopsis

## Abstract

DNA methylation is an important epigenetic modification required for the specific regulation of gene expression and the maintenance of genome stability in plants and animals. However, the mechanism of DNA demethylation remains largely unknown. Here, we show that two SGS3-like proteins, FACTOR OF DNA DEMETHYLATION 1 (FDDM1) and FDDM2, negatively affect the DNA methylation levels at ROS1-dependend DNA loci in Arabidopsis. FDDM1 binds dsRNAs with 5′ overhangs through its XS (rice gene X and SGS3) domain and forms a heterodimer with FDDM2 through its XH (rice gene X Homology) domain. A lack of FDDM1 or FDDM2 increased DNA methylation levels at several ROS1-dependent DNA loci. However, FDDM1 and FDDM2 may not have an additive effect on DNA methylation levels. Moreover, the XS and XH domains are required for the function of FDDM1. Taken together, these results suggest that FDDM1 and FDDM2 act as a heterodimer to positively modulate DNA demethylation. Our finding extends the function of plant-specific SGS3-like proteins.

## 1. Introduction

The methylation status of DNA at the 5′ position of cytosine (5 mC) plays crucial roles in plants’ developmental regulation and environmental adaptation by affecting gene expression and genome stability [1,2]. The proper level of DNA methylation is dynamically regulated by de novo methylation and the maintenance of methylation and demethylation [1,3,4]. Plant DNA methylation occurs in three cytosine contexts at CG, CHG, and CHH (where H represents A, T, or C) [5,6]. The de novo methylation is established by the RNA-directed DNA methylation (RdDM) pathway, in which DNA is methylated by DOMAINS REARRANGED METHYLTRANSFERASE 2 (DRM2) [7]. Once established, DNA methylation is maintained by a multitude of DNA methyltransferases, including METHYLTRANSFERASE 1 (MET1), CHROMOMETHYLASE 3 (CMT3), and CHROMOMETHYLASE 2 (CMT2), in a manner dependent on the cytosine sequence context [8,9,10]. In addition, DNA demethylation also contributes to methylation levels [11]. In Arabidopsis, four DNA glycosylases, including REPPESSOR OF SILENCING 1 (ROS1), TRANSCRIPTIONAL ACTIVATOR DEMETER (DME), DEMETER-LIKE PROTEIN 2 (DML2), and DML3, have been characterized to remove 5 mC from cytosines [12,13,14].

The plant-specific SGS3-like proteins have been reported to play essential roles in post-transcriptional gene silencing (PTGS) and RdDM [15,16,17,18,19,20,21]. The Arabidopsis genome encodes 14 SGS3-like proteins [18,22]. Among them, SGS3 is required for natural virus resistance and regulates the production of trans-activating small interfering RNAs [17,23]. INVOLVED IN DE NOVO DNA METHYLATION (IDN2, also known as RDM12) and its closely related proteins positively act in the RdDM pathway [15,16,20,21]. The SGS3-like proteins harbor various combinations of an XS domain required for RNA-binding, a zinc-finger domain, a coil-coil domain and an XH domain that mediates protein-protein interaction [17]. It is reported that IDN2 and FACTOR OF DNA METHYLATION 1 (FDM1) bind 5′ overhanging double-stranded RNAs (5′ dsRNAs) through the XS and coil-coil domains [15,18,21]. However, the biological function of other SGS3-like proteins remains unclear.

Here, we report that the other two SGS3-like proteins, FACTOR OF DNA DEMETHYLATION 1 (FDDM1, AT5G59390) and FDDM2 (AT4G01180), form a complex to participate in DNA demethylation in Arabidopsis. We found that FDDM1 binds dsRNAs with 5′ overhangs, which requires its XS domain, and that FDDM1 forms a heterodimer with FDDM2 through its XH domain. Loss-of-function mutations of FDDM1 and FDDM2 led to increased methylation levels of several examined loci. Interestingly, double mutant analyses showed that FDDM1 and FDDM2 do not work redundantly in modulating DNA methylation levels, indicating the FDDM1 and FDDM2 act as a heterodimer to promote DNA demethylation.

## 2. Materials and Methods

### 2.1. Plant Materials and Growth Condition

Salk_059303 (*fddm1-1*), Salk_021139 (*fddm1-2*), and CS822551 (*fddm2-1*) were obtained from the ABRC Stock Center and are in Columbia (Col) genetic background. These mutants were identified with a combination of gene-specific primers and T-DNA primers (Table S1), and the T-DNA insertion sites were confirmed by sequencing. The *fddm1-2 fddm2-1* mutant was generated through crossing single mutants. Plants were grown at 22 °C with 16 h light/8 h dark cycles in the growth chamber.

### 2.2. Construction of Plasmids and Plant Transformation

To generate *pFDDM1:FDDM1-GFP*, a 4.1 Kb genomic DNA fragment containing the *FDDM1* promoter and coding region was amplified and cloned into the *pMDC204* binary vector. *FDDM1-T1* (lacking the XH domain) and *FDDM1-T2* (lacking the XS domain) were cloned into the *pMDC83* vector to generate *35S:FDDM1-T1* and *35S:FDDM1-T2* constructs, respectively. The FDDM2 coding sequence was PCR amplified and inserted into the *pMDC83* vector to generate the *35S:FDDM2* construct. The primers used are listed in Table S1. All of the binary constructs were transformed into *fddm1-1* or *fddm2-1* mutants through Agrobacterium-mediated transformation. The transgenic plants were screened on a MS medium with Hygromycin B.

### 2.3. DNA Methylation Assay

The DNA methylation assay was performed as described previously [24]. The genomic DNAs were digested with a methylation-sensitive restriction enzyme and subsequently used for PCR analysis. The undigested genomic DNA was simultaneously amplified as the loading controls using primers listed in Table S1. For bisulfite sequencing, genomic DNA was converted with a BisulFlash DNA modification Kit (Epigentek) following the manual’s instructions. The targeting loci were PCR amplified and ligated into a *pGEM T* vector for DNA methylation analysis.

### 2.4. RT-PCR Analysis 

Total RNA was extracted from inflorescence with a TRIzol reagent and reverse-transcribed using oligo-dT primers with Promega M-MLV. The resulting cDNAs were used as templates for PCR amplification with gene-specific primers (Table S1).

### 2.5. DNA/RNA Binding Assay

The DNA/RNA binding assays were performed as previously described [18]. *FDDM1* and truncated *FDDM1* were amplified and cloned into a *pMAL-c5x* vector (NEB) to generate MBP fusion constructs. The MBP fusion proteins were expressed in *Escherichia coli* (*E. coli*) BL21 and purified as described [18]. The radioactive labeled ssRNA, dsRNAs, ssDNA, and dsDNAs were produced according to [18]. The DNA primers are listed in Table S1.

### 2.6. Yeast Two-Hybrid Assay

*FDDM1*, *FDDM1-T1*, and *FDDM1-T2* were cloned into *pGADT7* (AD) or *pGBKT7* (BD) (Clontech, Mountain View, CA, USA) to generate the various constructs used for the Yeast two-hybrid assay. The *BD-FDDM1-T3* and *BD-FDDM1-T4* constructs were generated using the PfuUltra II Fusion HS DNA polymerase (600670; Agilent, Santa Clara, CA, USA). *FDDM2* was cloned into *pGADT7* to generate the *AD-FDDM2* construct. The primers are listed in Table S1. The bait and prey pair constructs were co-transformed into yeast strain AH109. The interactions were tested on drop-out medium without tryptophan and leucine (–TL) or without adenine, histidine, tryptophan, and leucine (-AHTL). The interaction of the FDM1 XH domain with FDM1 itself was employed as a positive control [18].

### 2.7. BiFC and Co-IP Assays 

BiFC and co-IP assays were performed as previously described [25]. For BiFC, paired nVenus-FDDM1 and cCFP-FDDM1 or cCFP were co-expressed in *Nicotiana benthamiana* (*N. benthamiana*) leaves. After 40 h of expression, a fluorescence signal was detected using a confocal microscope (Fluoview 500 workstation; Olympus, Tokyo, Japan). To examine the interaction by co-IP assay, the combination of MYC-FDDM2 with FDDM1-GFP or GFP was co-expressed in *N. benthamiana* leaves. IP was carried out with protein extracts using anti-GFP antibodies, and the proteins were detected with the antibodies against YFP (B230720; Biolegend, San Diego, CA, USA) or MYC (06-340; Millipore, Burlington, MA, USA).

## 3. Results

### 3.1. XS Domain of FDDM1 Is Required for Binding of 5′ Overhang dsRNAs

**Figure 1:**
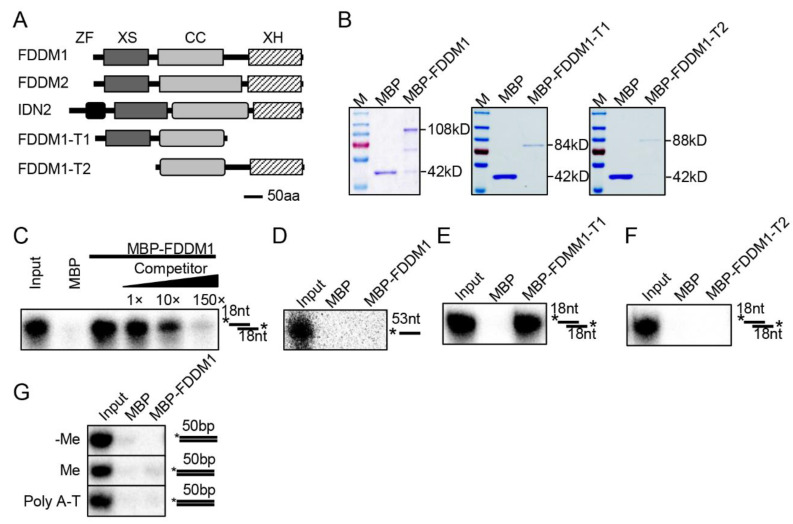
**FDDM1 binds 5′ overhang dsRNAs but not DNAs.** (**A**) The schematic structure of the FDDM1, truncated FDDM1, FDDM2 and IDN2 proteins. ZF: Zinc-finger; CC: Coil-Coil. (**B**) The purified proteins used in RNA and DNA binding assays. Proteins were resolved by SDS–PAGE gel and stained with Coomassie Brilliant Blue. The protein molecular masses are indicated on the right. (**C**,**D**) FDDM1 binds 5′ overhang dsRNA but not single-stranded RNA (ssRNA). The probes used in the binding assay are shown on the right. * indicates radioactive labeled RNA strand. 5′ overhang dsRNA: 35 bp dsRNA with 18 nt overhangs at each end. Competitor: unlabeled probe of the same sequence. (**E**,**F**) The XS domain, but not the XH domain, is required for the FDDM1-RNA interaction. (**G**) FDDM1 does not bind DNAs. Various DNA probes used in the binding assay are shown on the right. Asterisks indicate radioactive labeled DNA strand. Approximately 50 μg proteins were used for the binding assay.

FDDM1 (AT5g59390) and FDDM2 (AT4g01180) are two uncharacterized SGS3-like proteins that have high similarities (~82%) (Figure S1). They share high similarities with IDN2 and have the conserved domains of SGS3-like proteins, including XS, the coil-coil domain, and the XH domains (Figure 1A). As IDN2 binds 5′ overhanging dsRNAs (5′ dsRNAs) through its XS and coil-coil domains [15,21], we suspected that FDDM1/FDDM2 might also bind 5′ dsRNAs. We then used FDDM1 as a reporter to test this possibility through an RNA pull down assay. The recombined full-length and truncated FDDM1 fused with a maltose-binding protein epitope at their N terminus were expressed in *E. coli*, purified with amylose resin, and then incubated with radioactive labeled RNAs, including a single-stranded RNA and a 5′ dsRNA (Figure 1B–F). FDDM1 and FDDM1-T1 (lacking the XH domain) retained the 5′ dsRNAs but not the ssRNA (Figure 1C–E). In contrast, FDDM1-T2 (lacking the XS domain) did not bind the 5′ dsRNAs (Figure 1F). The addition of unlabeled 5′ dsRNA of the same sequence reduced the binding of the radioactive one (Figure 1C). In addition, we found that FDDM1 did not bind either methylated or unmethylated dsDNAs (Figure 1G). However, we cannot rule out that FDDM1 may bind other specific DNA sequences. These results demonstrate that FDDM1 binds 5′ dsRNAs, which requires the XS domain.

### 3.2. FDDM1 and FDDM2 Are Involved in DNA Demethylation

**Figure 2:**
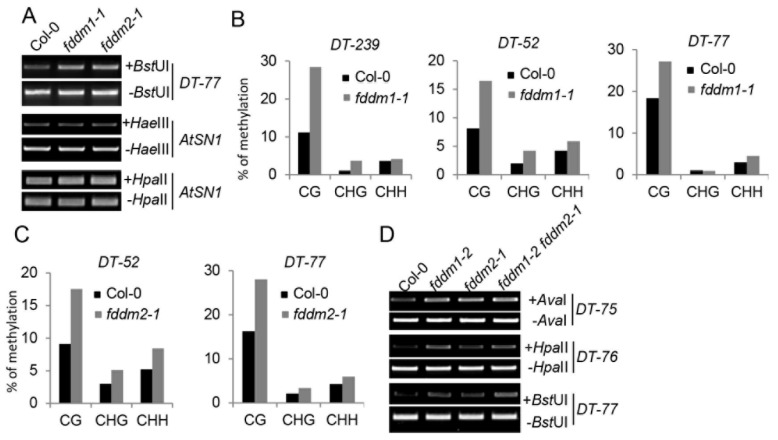
**FDDM1 and FDDM2 are required for DNA demethylation.** (**A**) FDDM1 and FDDM2 are required for DNA demethylation at the DT-77 locus. *Hae*III- or *Hpa*II-digested genomic DNAs were used for the PCR amplification of *AtSN1*, while *Bst*UI-treated genomic DNAs were used for the amplification of *DT-77*. Undigested genomic DNAs are used as loading controls. (**B**,**C**) The bisulfite sequencing analyses of DNA methylation at various genotypes. The percentage of methylated cytosine in different cytosine contexts is shown. (**D**) FDDM1 and FDDM2 do not act redundantly in DNA demethylation. Restriction enzyme-digested and undigested (loading control) DNAs were used as templates for the PCR amplification of various loci.

In order to identify the functions of FDDM1 and FDDM2, we obtained their T-DNA insertion null mutants from the Arabidopsis stock center, including Salk_059303 (*fddm1-1*), Salk_021139 (*fddm1-2*), and CS822551 (*fddm2-1*) (Figure S1A–D). Because FDDM1 and FDDM2 are homologs of IDN2, we first evaluated the effect of *fddm1* and *fddm2* on the DNA methylation status at the *ATSN1* locus, which is silenced in the wild-type plant. The loss-of-function mutations of FDDM1 and FDDM2 did not affect DNA methylation levels at the *ATSN1* locus (Figure 2A). Recent studies also indicate the involvement of non-coding RNAs in active DNA demethylation at a specific DNA locus [20]. Since FDDM1 and FDDM2 are RNA-binding proteins, we reasoned that they might function in the DNA demethylation process. We examined the DNA methylation status at the *DT-77* locus, whose methylation is controlled by the DNA demethylation enzyme ROS1 [20]. Relative to Col (wild-type plant; WT), the DNA methylation contents of *DT-77* were increased in *fddm1-1*, *fddm1-2*, and *fddm2-1* (Figure 2A and Figure S3A). We further analyzed the DNA methylation status at additional ROS1-dependent DNA loci, including *DT-239*, *DT-52*, and *DT-77*, using bisulfite sequencing. The DNA methylation contents were increased in *fddm1-1* and *fddm2-1* when compared with WT (Figure 2B,C). The expression of a wild-type copy of *FDDM1* and *FDDM2* in *fddm1-1* and *fddm2-1*, respectively, rescued the DNA methylation status at various DNA target loci (Figure S3B,C). These results suggest that FDDM1 and FDDM2 may modulate DNA methylation levels at ROS1-depedent DNA loci.

The high similarity between FDDM1 and FDDM2 suggests that they may act redundantly in modulating DNA methylation at ROS1-dependent DNA loci. We therefore constructed an *fddm1-2 fddm2-1* double mutant and examined the demethylation status in the various loci in this mutant. Unexpectedly, the DNA methylation contents in *fddm1-2 fddm2-1* seem similar to those in *fddm1-2* or *fddm2-1* (Figure 2D and Figure S3D), suggesting that FDDM1 and FDDM2 may not redundantly affect DNA methylation.

### 3.3. FDDM1 and FDDM2 Form a Heterodimer

**Figure 3:**
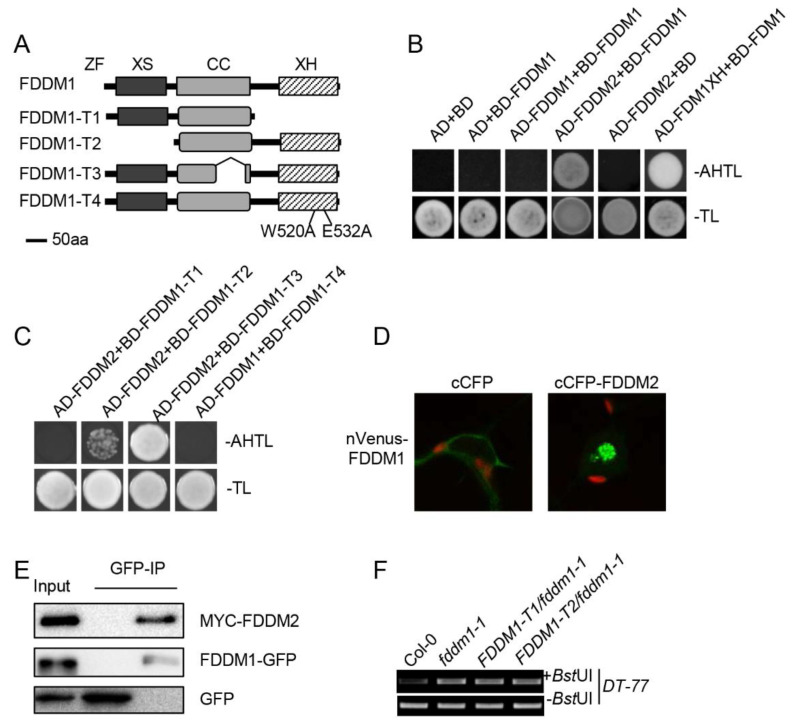
**FDDM1 and FDDM2 act in DNA demethylation via forming a heterodimer.** (**A**) The schematic structure of the truncated FDDM1 proteins. (**B**) FDDM1 interacts with FDDM2. Protein–protein interaction enables the growth of yeast cells on an adenine-deficient medium (–Ade–Leu–Trp). The interaction between FDM1XH and FDM1 was used as the positive control. (**C**) The XH domain is required for the FDDM1–FDDM2 interaction. (**D**) A BiFC analysis to detect the FDDM1–FDDM2 interaction in tobacco leaf cells. The green color indicates the BiFC signal (originally yellow fluorescence) detected by confocal microscopy. (**E**) A co-IP analysis to detect the FDDM1–FDDM2 interaction. MYC-FDDM2 co-expressed with FDDM1-GFP or GFP in tobacco leaves. IP was performed with anti-GFP antibodies, and the proteins were detected with the antibodies against MYC or GFP. (**F**) The XS and XH domains of FDDM1 are required for DNA demethylation. The restriction of enzyme-digested and undigested (loading control) DNAs were used as templates for the PCR amplification of *DT-77*.

The genetic non-redundancy of FDDM1 and FDDM2 raised a possibility that FDDM1 and FDDM2 act as a complex in DNA demethylation. We first used a yeast two-hybrid assay to test this possibility. The co-expression of BD-FDDM1/AD-FDDM2, but not the negative control pairs, enabled yeast cells to grow in the medium lacking Ade (Figure 3A,B), showing the FDDM1–FDDM2 interaction. In this assay, we employed the interaction between FDM1XH (XH domain of FDM1) and the full-length FDM1 as a positive control, which was reported by our lab previously [19]. We also did not observe the self-interaction of FDDM1 (Figure 3B). Next, we determined the protein domain of FDDM1 required for the FDDM1–FDDM2 interaction using truncated FDDM1 proteins. Deletion of the XH domain (FDDM1-T1), but not the XS domain (FDDM-T2) and the coil-coil domain (FDDM-T3), abolished the interaction (Figure 3C). Two conserved amino acids, W (tryptophan) and E (glutamic acid) in the XH domain, were reported to play the key roles in mediating protein interaction [19]. To validate our result, we replaced the two conserved amino acids (W520 and E532) with A (alanine) in the XH domain of FDDM1 (FDDM1-T4; Figure 3A). As expected, FDDM1-T4 did not interact with FDDM2 (Figure 3C). The results of the yeast two-hybrid indicate that the XH domain of FDDM1 mediates the interaction with FDDM2.

To validate the interaction observed in Y2H, we performed the bimolecular fluorescence complementation (BiFC) assay in tobacco leaf cells by transiently co-expressing nVenus-FDDM1 (FDDM1 fused with the N-terminal fragment of Venus) with cCFP-FDDM2 (FDDM2 fused with the C-terminal fragment of cyan fluorescent protein). The yellow fluorescence signal was detected in the nucleus when nVenus-FDDM1 co-expressed with cCFP-FDDM2, but not in the negative control cCFP (Figure 3D). Additionally, we also co-transformed the MYC-tagged FDDM2 with GFP-tagged FDDM1 or GFP in tobacco leaves and tested their interactions by coimmunoprecipitation (co-IP) assay. In the FDDM1-GFP, but not in the GFP precipitated fraction, MYC-FDDM2 protein was detected by western blot (Figure 3E). Taken together, these results reveal that FDDM1 and FDDM2 interact in plant cells.

### 3.4. XS and XH Domains of FDDM1 Are Required for Modulating DNA Methylation Levels

Based on the fact that the XS and XH domains play important roles in dsRNA binding and heterodimer formation, we wanted to know the effect of the XH and XS domains on the FDDM1 function in plants. We generated transgenic *fddm1-1* plants expressing *FDDM1-T1* (lacking the XH domain) or *FDDM1-T2* (lacking the XS domain). Unlike the full-length FDDM1 protein, FDDM1-T1 and FDDM1-T2 did not complement the increased DNA methylation levels in *fddm1-1* (Figure 3D and Figure S4). These results demonstrate that the XH domain and the XS domain are essential for the function of FDDM1 in DNA demethylation.

## 4. Discussion

In summary, we show that FDDM1 and FDDM2 participate in DNA methylation in Arabidopsis. This result adds a new function to the plant-specific SGS3-like gene family. FDDM1 and FDDM2 do not act redundantly. Rather, they likely form a hetero-complex to function, as a lack of the XH domain of FDDM1 disrupts the FDDM1-FDDM2 interaction, resulting in increased DNA methylation levels.

How does the FDDM1-FDDM2 complex affect DNA methylation? There are at least three possibilities. First, it may positively contribute to DNA demethylation through the ROS1 pathway. The IDN2 complex is proposed to bind the RNA duplex formed by siRNA and long non-coding RNAs to trigger downstream events in the RdDM pathway [16,18,20]. Thus, the FDDM1–FDDM2 complex may bind non-coding RNAs to facilitate DNA demethylation, given the fact that the FDDM1 mutant lacking the ability to bind 5′ dsRNAs fails to rescue the increased DNA methylation levels in *fddm1*. In addition, IDN2 has been shown to interact with chromatin-remodeling factors to contribute to RdDM [26]. By analog, FDDM1/FDDM2 may act similarly in modulating DNA demethylation. Second, it may be a negative regulator of DNA methylation process. However, the DNA methylation of AtSN1 is not affected by *fddm1*, suggesting that FDDM1 and FDDM2 may not function through the RdDM pathway. Third, it may indirectly contribute to the DNA methylation process through modulating the expression of genes involved in DNA methylation. Clearly, these possibilities need to be further examined.

## Supplementary Materials

The following are available online at https://www.mdpi.com/article/10.3390/genes13020339/s1, Figure S1: The amino acid sequence alignment of FDDM1 (AT5g59390) and FDDM2 (AT4g01180). Figure S2: Identification of fddm1-1, fddm1-2 and fddm2-1. Figure S3: FDDM1 and FDDM2 are required for DNA demethylation. Figure S4: FDDM1-T1 and FDDM-T2 proteins in transgenic plants detected by western blot. Table S1: Primers used in this study.
