# Supplementary material for: FDDM1 and FDDM2, Two SGS3-like Proteins, Function as a Complex to Affect DNA Methylation in Arabidopsis
Source: Genes (Basel). 2022 Feb 12;13(2):339. doi: 10.3390/genes13020339 (PMC8872474; doi:10.3390/genes13020339)
Supplement: Supplementary file 1 [file genes-13-00339-s001.zip › genes-1532273-supplementary.pdf]

Supplementary data

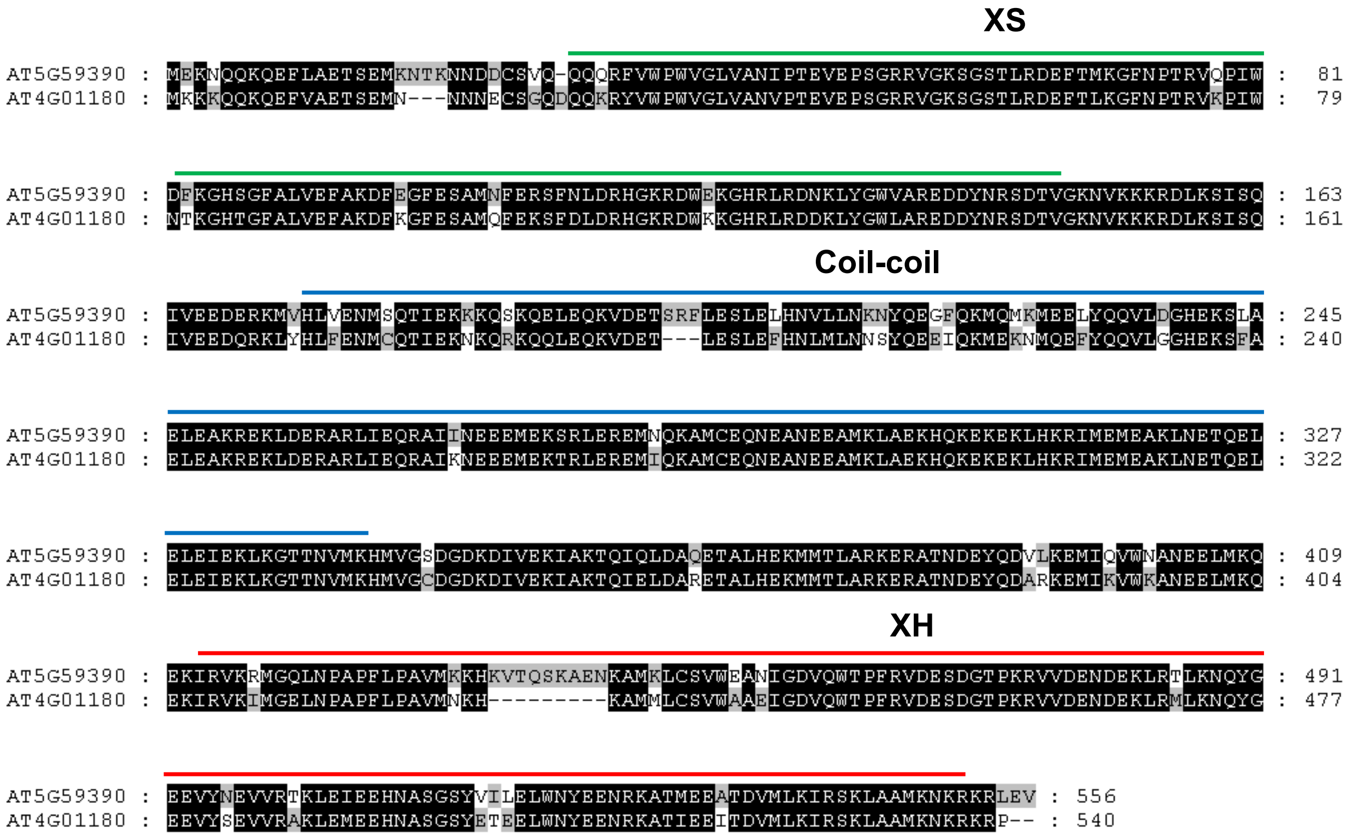

Figure S1. The amino acid sequence alignment of FDDM1 (AT5g59390) and FDDM2 (AT4g01180). The conserved domains of XS, Coil-coil, and XH were shown.

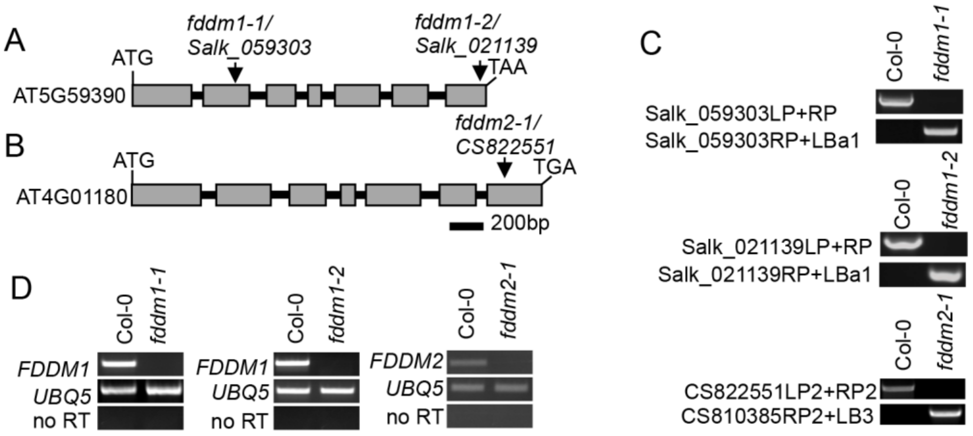

Figure S2. Identification of *fddm1-1, fddm1-2* and *fddm2-1*. (A) and (B) Schematic diagrams of gene structures and the T-DNA insertion positions. Gray boxes indicate the coding region and the lines indicate introns. (C) PCR analyses of T-DNA insertion in *fddm1-1*, *fddm1-2* and *fddm2-1*. Salk_021139LP/RP and Salk_059303LP/RP are gene specific primers for *FDDM1*. CS822551LP/RP are gene specific primers for *FDDM2*. LBa1 and LB3 indicate T-DNA specific primers. (D) The expression of *FDDM1* and *FDDM2* in various genotypes detected by RT-PCR. Amplification of *UBQ5* with or without reverse transcription was used as the control.

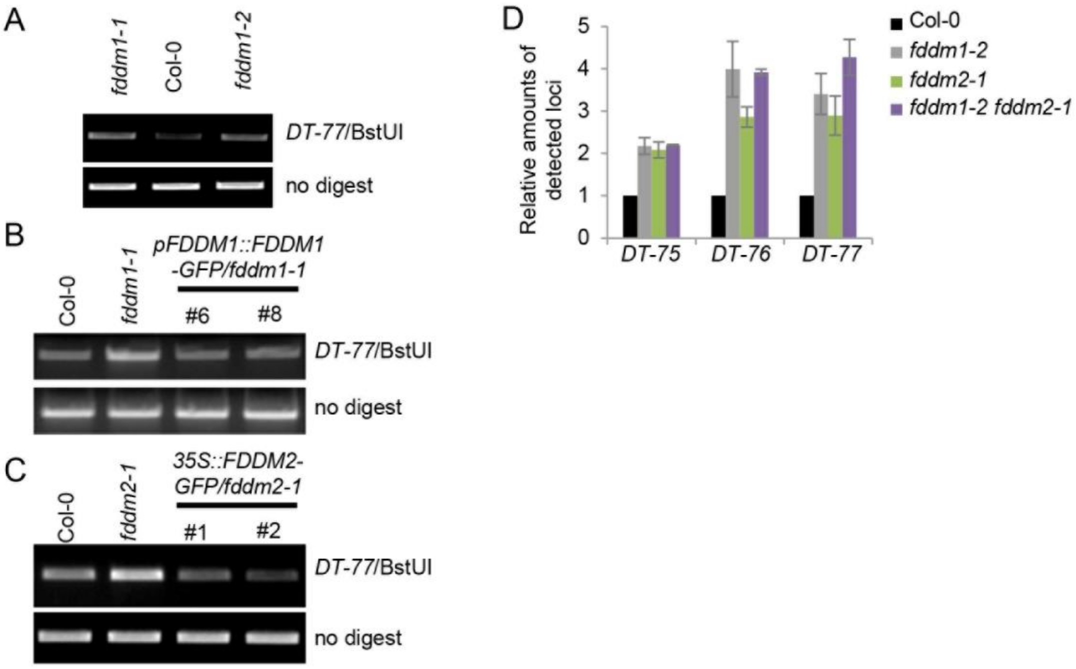

Figure S3. FDDM1 and FDDM2 are required for DNA demethylation. (A) DNA demethylation is impaired in *fddm1-1* and *fddm1-2*. (B) Expression of FDDM1 complements the defects of DNA demethylation in *fddm1-1*. (C) Expression of FDDM2 complements the defects of DNA demethylation in *fddm2-1*. *Bst*UI-treated genomic DNAs were used for the amplification of *DT-77*. Undigested genomic DNAs are used as loading controls. (D) Quantitative analysis of the gel bands shown in Figure 2D. The amounts of Col-0 were set as 1.

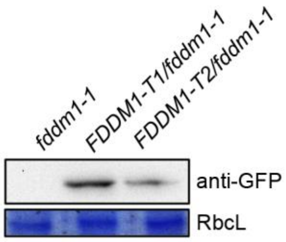

Figure S4. FDDM1-T1 and FDDM-T2 proteins in transgenic plants detected by western blot. Proteins were resolved in SDS-PAGE gel and detected by anti-GFP antibodies. Bands of Rubisco large subunit (RbcL) were shown as loading control.

Table S1. Primers used in this study.

Name	Sequence	Application
**Primers for T-DNA identification**		
CS822551LP	GTTTTCAAGCCTTGGGAGAAG	T-DNA
CS822551RP	TGTTCCAAACTCTTTGGCTTG	T-DNA
salk_059303RP	TCAAGGGTCATTCTGGTTTTG	T-DNA
salk_059303LP	GAGCGTCAAGCTGAATCTGAG	T-DNA
salk_021139LP	TTATCGAAGTGCAAGTACCCG	T-DNA
salk_021139RP	TGTGGGAAGCAAACATAGGAG	T-DNA
LBa1	TGGTTCACGTAGTGGGCCATCG	T-DNA
LB3	TAGCATCTGAATTTCATAACCAATCTCGATACAC	T-DNA
**Primers for constructs**		
FDDM1 (BamHI)-1F	CGGGATCCATATGGAGAAAAATCAACAAAAGC	AD-FDDM1
FDDM1 (SacI)-1R	ACGAGCTCTTAGACCTCAAGCCTCTTTCG	AD-FDDM1
FDDM1 (SalI)-1R	ACGCGTCGACTTAGACCTCAAGCCTCTTTCG	BD-FDDM1
FDDM1(SalI)-R3	ACGCGTCGACTTA AATATCTTTATCTCCATCACT	BD-FDDM1T1
FDDM1(BamH1)-F3	CGGGATCCAT ATGATTGTGGAGGAAGACGAGAGG	BD-FDDM1T2
FDDM1W520A-F	CATCACATCTGTTGCTGCCTCCATCGTCGCCTT	FDDM1 T4
FDDM1W520A-R	AAGGCGACGATGGAGGCAGCAACAGATGTGATG	FDDM1 T4
FDDM1E532A-F	CAGCTATGTGATTTTAGAGCTTGCGAACTATGAGGAAAACCGAAAG	FDDM1 T4
FDDM1E532A-R	CTTTCGGTTTTCCTCATAGTTCGCAAGCTCTAAAATCACATAGCTG	FDDM1 T4
FDDM1del312-342F	GAGAAGGAGAAGCTTCATCATATGGTGGGGAGTGAT	FDDM1 T3
FDDM1del312-342R	ATCACTCCCCACCATATGATGAAGCTTCTCCTTCTC	FDDM1 T3
FDDM2 (BamHI)-1F	CGGGATCCATATGAAGAAGAAACAACAAAAGC	AD-FDDM2
FDDM2 (SacI)-1R	ACGAGCTC TCAAGGCCTCTTTCGTTTGTTC	AD-FDDM2
FDDM1p-F	CACC CTATTACGAACCAAATACAGGA	FDDM1 genome amplification
FDDM1 35SF	CACCATGGAGAAAAATCAACAAAAGC	FDDM1 CDS amplification
FDDM1 35SR	GACCTCAAGCCTCTTTCGTTTG	FDDM1 CDS amplification
FDDM1(1-358)-R	AATATCTTTATCTCCATCACTCC	FDDM1 T1 construct

FDDM1(164-561)-F	CACC ATGATTGTGGAGGAAGACGAGAGG	FDDM1 T2 construct
FDDM2 35SF	CACCATGAAGAAGAAACAACAAAAG	FDDM2 CDS amplification
FDDM2 35SR	AGGCCTCTTTCGTTTGTTCTTC	FDDM2 CDS amplification
FDDM1-MBP F	AT GCGGCCGC ATGGAGAAAAATCAACAAAAGC	MBP-FDDM1 construct
FDDM1-MBP R3	ATGTCGAC TTAGACCTCAAGCCTCTTTCG	MBP-FDDM1 construct
FDDM1T1-MBP R4	ATGTCGAC TTAAATATCTTTATCTCCATCACT	MBP-FDDM1T1 construct
FDDM1T2-MBP F2	ATGCGGCCGCATGATTGTGGAGGAAGACGAGAGG	MBP-FDDM1T2 construct
**Primers for RT-PCR**		
RT-FDDM1-F	TGTGGGAAGCAAACATAGGAG	RT-PCR
RT-FDDM1-R	CTCCGGTATTGTTACTTGAATTA	RT-PCR
RT-FDDM2-F	GTTTTCAAGCCTTGGGAGAAG	RT-PCR
RT-FDDM2-R	TGTTCCAAACTCTTTGGCTTG	RT-PCR
N_UBQ5	GGTGCTAAGAAGAGGAAGAAT	RT-PCR
C_UBQ5	CTCCTTCTTTCTGGTAAACGT	RT-PCR
**Primers for DNA methylation analysis**		
DT-75-F	CATGTTGGAAATTTTGTACCCG	Chop-PCR
DT-75-R	GAGTATTATGAAGGAGCTCTACGA	Chop-PCR
DT-76-F	GAACCTGAGTGCTGAAGGTTACTC	Chop-PCR
DT-76-R	CAAGATTCAATACTTTACGACATG	Chop-PCR
DT-77-F	TGACCTGCATAGGCTATAACACA	Chop-PCR
DT-77-R	ATTGGAATCAATCCGAGTGG	Chop-PCR
AtSN1_HaeIII F	AGGATTTATTTCAATCCACGAACCTC	Chop-PCR
AtSN1_HaeIII R	CGACTCCCATAAGTAACGAGTTG	Chop-PCR
DT-239-F	CAAAAACAAATAATCCACCCTTACC	Chop-PCR, Bisulfite sequencing
DT-239-R	GATTATTGTAGGTGGTAAAATGGATAG	Chop-PCR, Bisulfite sequencing
At1g26400/DT-77-F2	GTAGTTTGAGATGATTAATGATAGAGTT	Bisulfite sequencing

At1g26400/DT-77-R2	AAACTTATTCAATCTTCAATACTCTAC	Bisulfite sequencing
DT-52-F1	GGTAGAGAGTTGGTTTGGTATTG	Bisulfite sequencing
DT-52-R1	CTAATCCTCATCCTCATCCTCATC	Bisulfite sequencing
**Primers for RNA/DNA binding**		
RNA1F	TAATACGACTCACTATAGGGCAACAACTGTCTCTTGGATCGT	Amplification of DNA template for RNA 1 and RNA 3
RNA1R	TGAGGAGCTCAACACATAAATGGCAATA	Amplification of DNA template for RNA 1
RNA2F	GATCGTATATTGCCATTTATGT	Amplification of DNA template for RNA 2
RNA2R	TAATACGACTCACTATAGGGGACATGGAGCTGGAGTGAGGAG	Amplification of DNA template for RNA 2
RNA3R	TAATACGACTCACTATAGGGTGAGGAGCTCAACACATAAATG	Amplification of DNA template for RNA 3
DNA-Me_S1	GCTCGTAGCTAACGAGCTCGACTCGTTGACATAGGCCATGGCGTAGACTC	unmethylated DNA probe
DNA-Me_S2	GAGTCTACGCCATGGCCTATGTCAACGAGTCGAGCTCGTTAGCTACGAGC	unmethylated DNA probe
DNA+Me_S1	GCTmCGTAGCTAAmCGAGCTmCGACTmCGTTGACATAGGCCATGGmCGTAGACTC*	methylated DNA probe
DNA+Me_S2	GAGTCTAmCGCCATGGCCTATGTCAAmCGAGTmCGAGCTmCGTTAGCTAmCGAGC	methylated DNA probe

*: mC indicates methylated C.
